# Combined treatment with DPP-4 inhibitor linagliptin and SGLT2 inhibitor empagliflozin attenuates neointima formation after vascular injury in diabetic mice

**DOI:** 10.1016/j.bbrep.2019.100640

**Published:** 2019-04-19

**Authors:** Hiroyuki Takahashi, Takashi Nomiyama, Yuichi Terawaki, Takeshi Horikawa, Takako Kawanami, Yuriko Hamaguchi, Tomoko Tanaka, Ryoko Motonaga, Takashi Fukuda, Makito Tanabe, Toshihiko Yanase

**Affiliations:** Department of Endocrinology and Diabetes Mellitus, School of Medicine, Fukuoka University, Fukuoka, Japan

**Keywords:** DPP-4I, SGLT2I, Neointima formation, VSMC proliferation

## Abstract

Incretin therapy has emerged as one of the most popular medications for type 2 diabetes. We have previously reported that the dipeptidyl peptidase-4 (DPP-4) inhibitor linagliptin attenuates neointima formation after vascular injury in non-diabetic mice. In the present study, we examined whether combined treatment with linagliptin and the sodium glucose cotransporter 2 (SGLT2) inhibitor empagliflozin attenuates neointima formation in diabetic mice after vascular injury. Diabetic *db/db* mice were treated with 3 mg/kg/day linagliptin and/or 30 mg/kg/day empagliflozin from 5 to 10 weeks of age. Body weight was significantly decreased by empagliflozin and the combined treatment. Blood glucose levels and glucose tolerance test results were significantly improved by empagliflozin and the combined treatment, but not by linagliptin. An insulin tolerance test suggested that linagliptin and empagliflozin did not improve insulin sensitivity. In a model of guidewire-induced femoral artery injury in diabetic mice, neointima formation was significantly decreased in mice subjected to combined treatment. In an *in vitro* assay using rat aortic smooth muscle cells (RASMC), 100, 500, or 1000 nM empagliflozin significantly decreased the RASMC number in a dose-dependent manner. A further significant reduction in RASMC proliferation was observed after combined treatment with 10 nM linagliptin and 100 nM empagliflozin. These data suggest that combined treatment with the DPP-4 inhibitor linagliptin and SGLT2 inhibitor empagliflozin attenuates neointima formation after vascular injury in diabetic mice *in vivo* and smooth muscle cell proliferation *in vitro*.

## Highlights

• Linagliptin and empagliflozin attenuate neointima formation in diabetic mice.
• Linagliptin and empagliflozin attenuate VSMC proliferation *in vitro*.
• Linagliptin and empagliflozin attenuate VSMC DNA synthesis without apoptosis.

## 1 Introduction

Recently, the vascular protective effect of anti-diabetic agents has been receiving much attention. Several newly identified anti-diabetic agents, such as sodium-glucose cotransporter 2 (SGLT2) inhibitors, have demonstrated reductions in cardiovascular (CV) events compared with placebo in large randomized control trials [1,2]. However, it has been reported that sitagliptin [3] and alogliptin [4] decrease cervical vascular thickness in Japanese patients with type 2 diabetes mellitus (T2DM). In addition, the dipeptidyl peptidase-4 (DPP-4) inhibitor linagliptin reduced CV events in a phase III program meta-analysis [5]. Because of their efficacy, safety, and tolerability, DPP-4 inhibitors have become one of the most popular anti-diabetic agents in Japan [6]. Furthermore, we have demonstrated that the glucose reduction caused by DPP-4 inhibitors increases in patients with a low body mass index and short duration of T2DM [7].

In addition to primary CV events, restenosis after coronary angioplasty is still a critical problem for patients with T2DM, even though high performance drug-eluting stents have been developed [8]. A guide-wire induced endothelial denudation injury is established as an experimental mouse model for restenosis and vascular thickening after coronary angioplasty, vascular injury, or vascular inflammation [9]. The pathogenesis of vascular thickening caused by wire injury mainly reflects vascular smooth muscle cells (VSMC) proliferation after phenotype switching of VSMCs [10]. Previously, we reported that NR4A neuron-derived orphan receptor1 (NOR1) has an important role in VSMC proliferation and neointima formation after vascular injury [11,12]. Furthermore, we found that anti-diabetic agent GLP-1 receptor agonist exenin-4 decreases vascular injury-induced neointima formation [13] via inhibition of NOR1 expression [14] independent of the glucose-lowering effect. In addition, we have previously reported the vascular protective effect of DPP-4 inhibitor linagliptin [15]. Linagliptin decreases neointima formation after vascular injury and attenuates VSMC proliferation independent of the glucose-lowering effect [15]. In our previous reports, we demonstrated the reduction of vascular thickening by incretin therapy, a GLP-1 receptor agonist, and DPP-4 inhibitor using a non-diabetic model to examine their vascular protective effects independent of glucose-lowering effects. In the current study, we examined the vascular protective effect of linagliptin in diabetic *db/db* mice. Furthermore, we examined the effects of combined treatment with linagliptin and the SGLT2 inhibitor empagliflozin.

## 2 Materials and methods

### 2.1 Animals

The study protocol was reviewed and approved by the Animal Care and Use Committee of Fukuoka University. The investigation conformed to the *Guide for the Care and Use of Laboratory Animals* published by the US National Institutes of Health (NIH Publication No. 85-23, revised 1996). Four-week-old male *db/db* mice were purchased from Charles River Laboratories Japan, Inc. (Yokohama, Japan). All mice were housed in polycarbonate cages with a wooden chip mat on the floor. Water was available *ad libitum*. *db/db* mice were divided into the following treatment groups: control (n = 7), linagliptin (n = 8), empagliflozin (n = 10), and linagliptin + empagliflozin (n = 9). Linagliptin and empagliflozin were kindly provided by Boehringer Ingelheim Pharma GmbH & Co. KG (Biberach an der Riss, Germany). At 5 weeks of age, control mice were fed normal chow (22.6% protein, 53.8% carbohydrate, 5.6% fat, 6.6% minerals, a vitamin mixture, and 3.3% fiber; 356 kcal/100 g) with the vehicle (control), and linagliptin-treated mice were fed normal chow with linagliptin (0.083 g/kg chow, resulting in mean plasma levels of 50–150 nM, corresponding to an oral dose of 3 mg/kg/day). Empagliflozin was dissolved in water and administered to the relevant experimental groups (30 mg/kg/day). The animal room had a 12-h light/dark cycle, constant temperature (22 ± 1 °C), and relative humidity of 55 ± 5% throughout the experimental period. Endothelial denudation injuries were induced in the left femoral artery at 6 weeks of age, followed by evaluation of neointima formation at 10 weeks of age.

### 2.2 Guidewire-induced endothelial denudation injury

A femoral artery endothelial denudation injury was established in *db/db* mice treated with the control, linagliptin (3 mg/kg/day), empagliflozin (30 mg/kg/day), or linagliptin + empagliflozin at 6 weeks of age, as described previously [[12], [13], [14], [15]]. Briefly, endovascular injury was induced by four passages of a 0.25-mm SilverSpeed-10 hydrophilic guidewire (Micro Therapeutics Inc., Irvine, CA, USA) into the left femoral artery. Mice were euthanized at 4 weeks after injury, and femoral arteries were isolated for analysis.

### 2.3 Glucose and insulin tolerance tests

A glucose tolerance test (GTT) was performed at 10 weeks of age, and an insulin tolerance test (ITT) was performed at 11 weeks of age. In the GTT, overnight-fasted mice were administered an intraperitoneal injection of 1 g glucose/kg body weight. Blood glucose levels were measured at 0, 15, 30, 60, and 120 min after the glucose injection. In the ITT, the mice were administered an intraperitoneal injection of 1 U insulin/kg body weight after 3 h of fasting. Blood glucose was monitored at 0, 15, 30, and 60 min after insulin injection. Insulin sensitivity was estimated by the percentage change in the plasma glucose concentration.

### 2.4 Tissue preparation and morphometry

Following sacrifice, mice were perfused via a cannula in the left ventricle with phosphate-buffered saline for 5 min, followed by 4% paraformaldehyde for 30 min at 100 cm H_2_O. The femoral arteries were embedded in paraffin, cut into 5-μm sections, and prepared for Elastica van Gieson staining. Serial sections of the 1 mm proximal region from the incision site of the wire insertion were evaluated using an Elastica van Gieson stain kit (HT25A-1KT; Sigma-Aldrich, Tokyo, Japan) to visualize the internal elastic lamina, as described previously [14,15]. Specimens were viewed under a BZ9000 microscope (Keyence, Tokyo, Japan). The intimal and medial areas were measured by computerized morphometry using a BZ-II analyzer (Keyence). Intimal hyperplasia was defined as the formation of a neointimal layer medial to the internal elastic lamina. The medial area represents the area between external and internal elastic laminas. The intima-to-media ratio was calculated as the intimal area divided by the media area, as described previously [[12], [13], [14], [15]].

### 2.5 Cell culture

Rat aortic smooth muscle cells (RASMC) were purchased from Lonza (Allendale, NJ, USA) and maintained in D-MEM/Ham's F-12 (042-30555, Wako, Osaka, Japan) supplemented with 10% fetal bovine serum and 1% penicillin/streptomycin. Cells were used between passages three and six for experiments, and individual experiments were repeated at least three times with different cell preparations.

### 2.6 Proliferation assay

Cell proliferation assays were performed as described previously [15] with minor modifications. Briefly, RASMC were seeded in 12-well tissue culture plates and maintained in complete media with or without 10 nM linagliptin and/or 100, 500, or 1000 nM empagliflozin. Cell proliferation was analyzed after 0–4 days by cell counting using a hemocytometer.

### 2.7 Reverse transcription (RT) and quantitative real-time PCR

RT and quantitative real-time PCR were performed as described previously [[16], [17], [18]]. Total mRNA from RASMCs was isolated using an RNeasy Mini Kit (Qiagen, Venlo, the Netherlands) and reverse transcribed into cDNA. Rat kidney total RNA and adrenal gland total RNA were purchased from Clontech (Palo Alto, CA, USA) and reverse transcribed into cDNA in the same manner. PCRs were performed using a Light Cycler 2.0 (Roche, Basel, Switzerland) and SYBR Premix Ex Taq™ II (Takara, Otsu, Japan). Each sample was analyzed in triplicate and normalized against *glyceraldehyde-3-phosphate dehydrogenase* (*GAPDH*) mRNA expression. The primer sequences were as follows: Rat *GAPDH*, 5′-TGAACGGGAAGCTCACTGG-3′ (forward), 5′-TCCACCACCCTGTTGCTGTA-3′ (reverse); Rat *SGLT1*, 5′-TGGCCATTTTCATCCTGCTG-3′ (forward), 5′-ACTTCTCGGAAAGCAAACCC-3′ (reverse); Rat *SGLT2*, 5′-AGGCATGATTAGCCGCATTC-3′ (forward), 5′-TTGGGCATGAGCTTCACAAC-3′ (reverse).

### 2.8 Apoptosis assay

To label nuclei of apoptotic cells, 1 × 10^5^ RSMC were plated on glass coverslips in Lab-Tek Chamber Slides (177380; Nunc, Thermo Scientific, Waltham, MA, USA) and maintained in complete media with 10 nM linagliptin, 500 nM empagliflozin or both for 48 h. The cells were fixed in 4% paraformaldehyde for 25 min. Terminal deoxynucleotidyl transferase-mediated dUTP nick end labeling (TUNEL) was performed using the DeadEnd Fluorometric TUNEL System (Promega, Madison, WI, USA), in accordance with the manufacturer's protocol. Cells treated with 1 U/100 μl RQ1 RNase-Free DNase (M6101; Promega) for 24 h were used as a positive control.

### 2.9 BrdU assay

To evaluate the cell proliferation of RASMCs, a bromodeoxyuridine (BrdU) incorporation assay was performed using a Cell Proliferation ELISA kit (1647229; Roche Applied Science, Mannheim, Germany). Briefly, RASMC were seeded at 4000 cells/well in 96-well culture plates in complete media (n = 5). At 60%–70% confluence, cells were treated with 10 nM linagliptin, 500 nM empagliflozin, or both for 48 h. A BrdU (10 μM) solution was added during the last 2 h of treatment. Next, the cells were dried and fixed, and the cellular DNA was denatured with FixDenat solution (Roche Applied Science) for 30 min at room temperature. A peroxidase-conjugated mouse anti-BrdU monoclonal antibody (Roche Applied Science) was added to the culture plates, followed by incubation for 90 min at room temperature. Finally, tetramethylbenzidine substrate was applied for 15 min at room temperature, and the absorbance of the samples was measured using a microplate reader at 450–620 nm. Mean data are expressed as a ratio of the control (untreated) cell proliferation.

### 2.10 Statistical analysis

Results are expressed as the mean ± SEM. All statistical analyses were performed using GraphPad Prism software (version 7.0; GraphPad Software, La Jolla, CA, USA). In experiments comparing multiple groups, differences were analyzed by one-way or two-way ANOVA, followed by Sidak's *post-hoc* test as appropriate. *P* < 0.05 was considered to be statistically significant.

## 3 Results

### 3.1 Combined treatment with linagliptin and empagliflozin attenuates neointima formation in diabetic mice

**Fig. 1:**
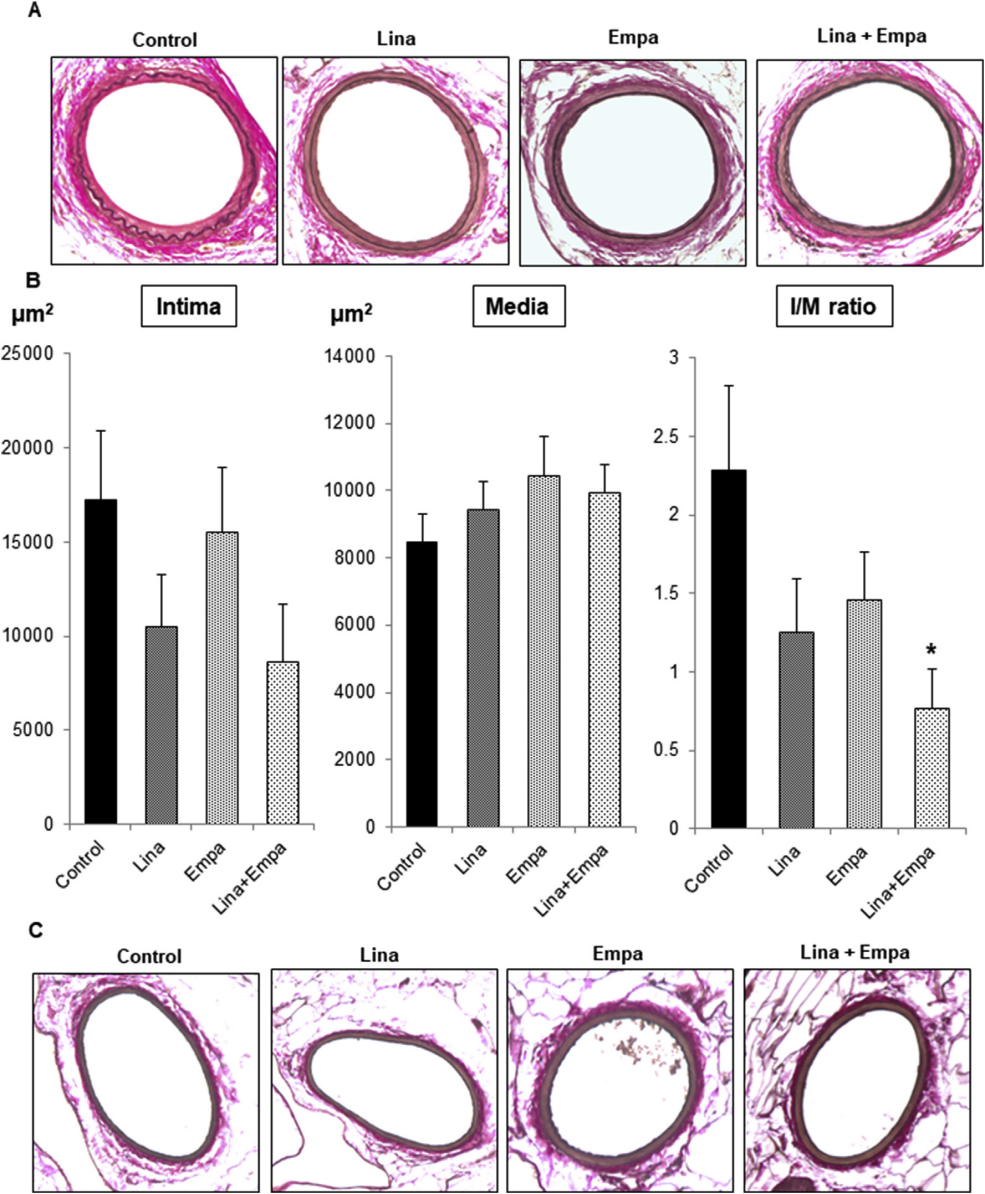
**Combined treatment with linagliptin and empagliflozin attenuates neointima formation after vascular injury in *db/db* mice.** Endothelial denudation injuries were induced in the left femoral artery of mice in control (n = 7), linagliptin (n = 8), empagliflozin (n = 10), and combined treatment groups (n = 9). The left femoral artery with injury (A) and right femoral artery without injury (B) were evaluated by staining with Elastica van Gieson to visualize the internal elastic lamina (magnification, × 200). (C) Intima and media areas, and the intima/media ratio were calculated for each group. Data are the mean ± SEM. One-way ANOVA was performed to calculate statistical significance (*P < 0.05 vs. control).

To evaluate neointima formation after vascular injury by endothelial denudation, we performed elastic staining as depicted in Fig. 1A. In the control vessel, clearly thickened neointima formation was observed. However, a much smaller neointima was observed in vessels from drug-treated mice. Intima and media area measurements revealed that single treatments with linagliptin or empagliflozin decreased neointima formation, although it was not statistically significant (Fig. 1B). However, combined treatment with linagliptin and empagliflozin significantly attenuated neointima formation compared with the control (Fig. 1B). In contrast, the right vessel with sham surgery did not change after left vessel injury and linagliptin and/or empagliflozin treatment (Fig. 1C), which is consistent with our previous report [15].

**Fig. 2:**
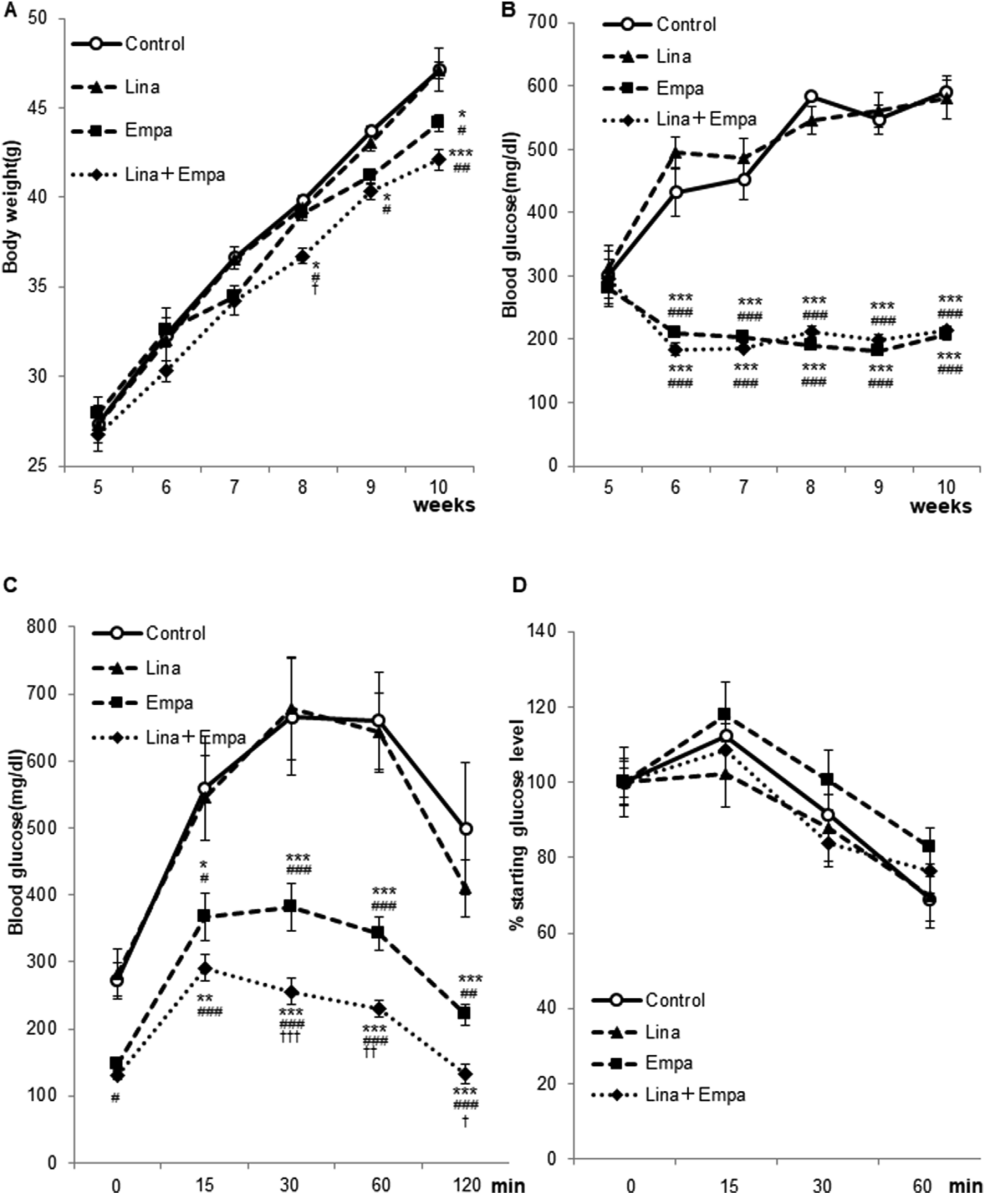
**SGLT2 inhibitor empagliflozin, attenuates body weight gain and improves glucose tolerance in *db/db* mice.** Comparison of the change in body weight (A) and casual blood glucose (B) in control (n = 7), linagliptin (n = 8), empagliflozin (n = 10), and combined treatment groups (n = 9). A GTT (C) was performed at 10 weeks of age, and an ITT (D) was performed at 11 weeks of age. Data are the mean ± SEM. Two-way repeated measures ANOVA was performed to calculate statistical significance (*P < 0.05, **P < 0.01, ***P < 0.001 vs. Control; ^#^*P* < 0.05, ^##^*P* < 0.01, ^###^*P* < 0.001 vs. linagliptin; ^†^*P* < 0.05, ^††^*P* < 0.01, ^†††^*P* < 0.001 vs. empagliflozin).

During the treatment period, we measured the blood glucose level and body weight of mice. The body weight was significantly decreased by empagliflozin and the combined treatment, but not by linagliptin (Fig. 2A). Although the blood glucose level was not decreased by linagliptin in *db/db* mice, a profound reduction in the blood glucose level was observed after empagliflozin and combined treatments (*P* < 0.05). Consistent with the blood glucose level, the GTT revealed that empagliflozin and the combined treatment, but not linagliptin, improved the glucose response curve (Fig. 2C). However, insulin sensitivity examined by the ITT was not changed by empagliflozin, linagliptin, or the combined treatment (Fig. 2D).

### 3.2 Combined treatment with linagliptin and empagliflozin attenuates VSMC proliferation *in vitro*

**Fig. 3:**
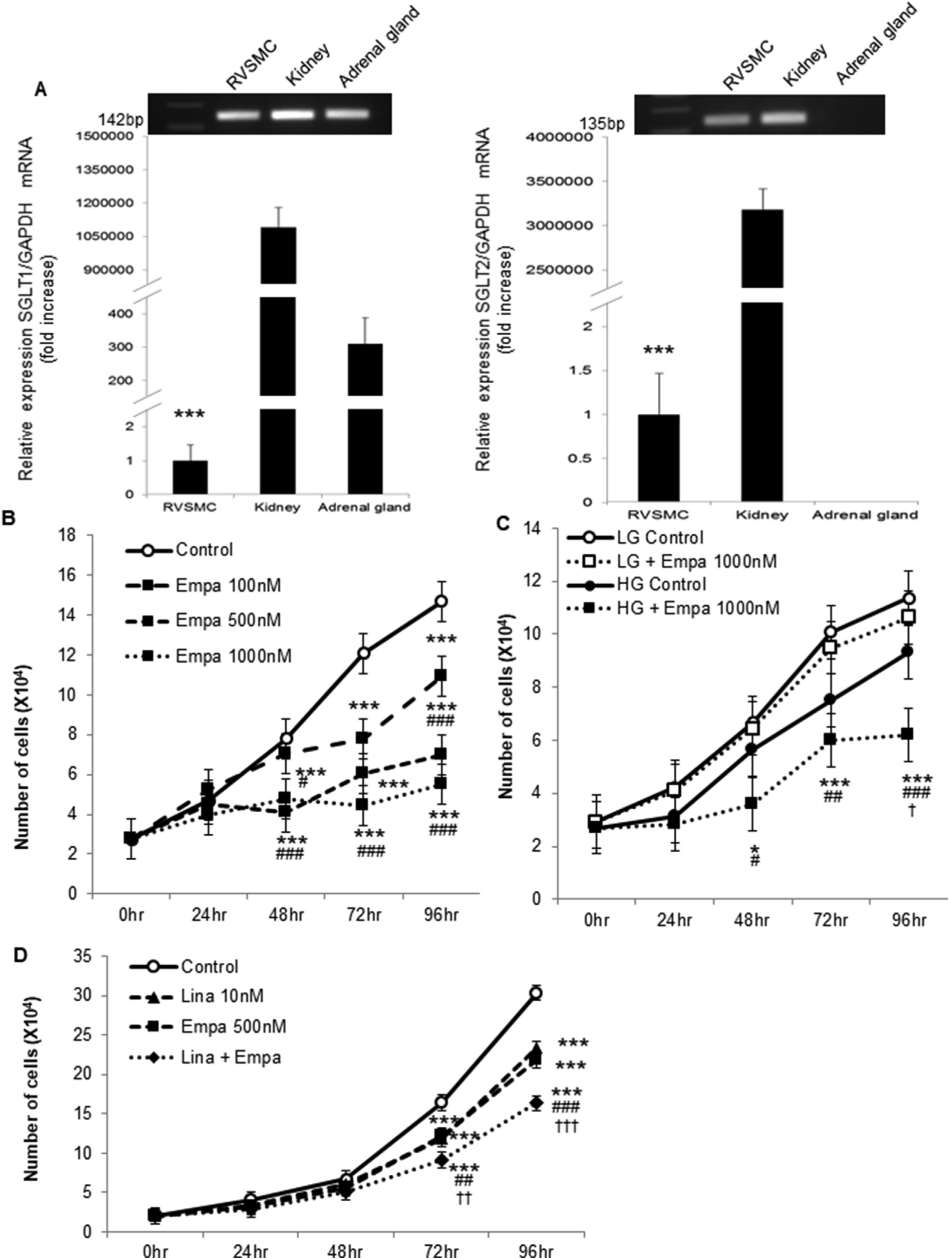
**Combined linagliptin and empagliflozin attenuates VSMC proliferation *in vitro*.** (A) RASMCs expressed *SGLT1* (left) and *SGLT2* (right) mRNAs (***P < 0.001 vs. control). (B) RASMCs were maintained in medium supplemented with 10% FBS and 17.5 mM d-glucose with the control (DMSO) or 100–1000 nM empagliflozin. After 0, 24, 48, 72, and 96 h, the cells were harvested, and cell proliferation was analyzed by cell counting using a hemocytometer. Two-way ANOVA was performed to calculate statistical significance (*P < 0.05, **P < 0.01, ***P < 0.01 vs. control; ^#^*P* < 0.05, ^##^*P* < 0.01 vs. 100 nM empagliflozin). (C) RASMCs were maintained in medium supplemented with 5.5 mM (LG) or 17.5 mM (HG) d-glucose with the control (DMSO) or 1000 nM empagliflozin. After 0, 24, 48, 72, and 96 h, the cells were harvested, and cell proliferation was analyzed. Two-way ANOVA was performed to calculate statistical significance (*P < 0.05, **P < 0.01, ***P < 0.01 vs. LG control; ^#^*P* < 0.05, ^##^*P* < 0.01 vs. LG + empagliflozin; ^†^*P* < 0.05 vs. HG control). (D) RASMCs were maintained in medium supplemented with 10% FBS and 17.5 mM d-glucose with 10 nM linagliptin, 500 nM empagliflozin, or both. After 0, 24, 48, 72, and 96 h, the cells were harvested, and cell proliferation was analyzed. Two-way ANOVA was performed to calculate statistical significance (*P < 0.05, **P < 0.01, ***P < 0.01 vs. Control; ^#^*P* < 0.05, ^##^*P* < 0.01 vs. linagliptin; ^††^*P* < 0.01, ^†††^*P* < 0.001 vs. empagliflozin).

We next examined the expression of *SGLT1* and *2* in VSMC using RASMC. Compared with the kidney (positive control), the expression of these genes was significantly lower in RASMCs, although it was detected (Fig. 3A). Additionally, expression of *SGLT1*, but not *SGLT2*, was detected in the adrenal gland (Fig. 3A), as reported previously [16], thus validating our experiments.

Following our previous report, which demonstrated that linagliptin attenuates VSMC proliferation *in vitro* [15], we next treated RASMC with empagliflozin. As shown in Fig. 3B, empagliflozin also significantly attenuated VSMC proliferation in a dose-dependent manner. Interestingly, the attenuation of VSMC proliferation by empagliflozin was observed in high glucose medium, but not in low glucose medium (Fig. 3C). These data suggest that empagliflozin attenuated VSMC proliferation via inhibition of glucose incorporation through SGLT2. As shown in Fig. 3D, both linagliptin and empagliflozin attenuated VSMC proliferation significantly, and further additive significant attenuation was observed by the combined treatment, which was consistent with the *in vivo* experiment (Fig. 1A and B).

### 3.3 Linagliptin and empagliflozin attenuate DNA synthesis, but do not induce apoptosis

**Fig. 4:**
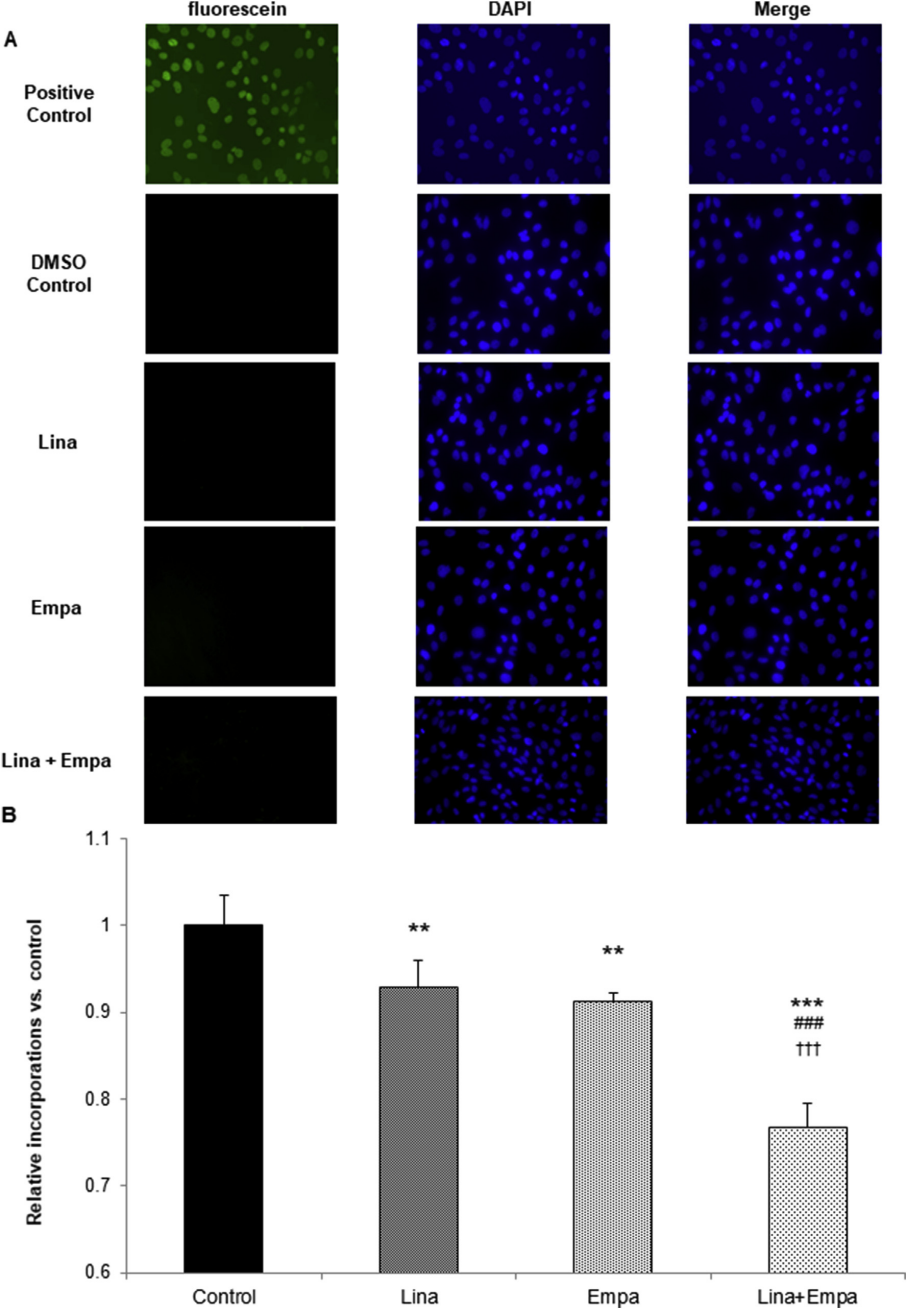
**Linagliptin and empagliflozin attenuate RASMC proliferation by reducing DNA synthesis and not by inducing apoptosis.** (A) RASMCs were plated on glass coverslips in Lab-Tek 2 well Chamber Slides. After incubation with 10 nM linagliptin, 500 nM empagliflozin, or both for 48 h, or with 1 U/100 μL RQ1 DNase (positive control) for 10 min, apoptotic cells were detected by TUNEL staining. Images shown are representative of three independent experiments. (B) RASMCs were seeded at a density of 4000 cells/well in 96-well plates in medium supplemented with 10% FBS and incubated with the control (DMSO), 10 nM linagliptin, 500 nM empagliflozin, or both for 48 h. A BrdU solution was added during the last 2 h, and cells were harvested for measurement of DNA synthesis using a microplate reader at 450–620 nm. One-way ANOVA was performed to calculate statistical significance (**P < 0.01, ***P < 0.001 vs. control; ^###^*P* < 0.001 vs. linagliptin; ^†††^*P* < 0.001 vs. empagliflozin).

Finally, we examined whether linagliptin and empagliflozin decrease VSMC proliferation by inducing apoptosis or attenuating DNA synthesis. As depicted in Fig. 4A, TUNEL-positive cells were not detected after treatment with linagliptin, empagliflozin, or both, suggesting that apoptosis was not induced in VSMCs. However, the BrdU incorporation assay revealed that linagliptin and empagliflozin significantly decreased BrdU incorporation (Fig. 4B). In addition, further significant reduction was observed after combined therapy with linagliptin and empagliflozin. These data suggested that linagliptin and empagliflozin decreased VSMC proliferation *in vitro* by additively attenuating VSMC DNA synthesis and not by inducing apoptosis.

## 4 Discussion

Recently, the aim of treating patients with diabetes has become not only glucose lowering, but also decreasing CV events. Several large randomized, placebo-controlled trials have suggested that the vascular-protective effect of anti-diabetic agents is dependent and independent of the glucose lowering effect. DPP-4 inhibitors have become one of the major anti-diabetic agents, especially in Japan [6], because of their efficacy and safety, as we have reported previously [7]. Among DPP-4 inhibitors, linagliptin is a unique compound with a xanthine structure. We have reported that linagliptin attenuates neointima formation after vascular injury and VSMC proliferation via its anti-oxidative stress effect, upregulation of the serum GLP-1 level, and its direct action on VSMC independent of its glucose-lowering effect [15]. In addition, another report has suggested that linagliptin improves high glucose-induced impairment of endothelium-dependent vascular relaxation by antioxidant activity independent of the glucose-lowering effect [17]. Based on these findings, CARMELINA^®^ demonstrated the cardiovascular and renal safety of linagliptin in diverse patients with T2DM [18]. Additionally, empagliflozin has been shown to significantly decrease CV events by EMPA-REG OUTCOME^®^ [1]. Using animal models, the mechanism by which empagliflozin induces vascular protection has been reported to involve reduction of oxidative stress and glucose toxicity [19], and improvement of inflammation and insulin sensitivity [20]. In the CANVAS program, SGLT2 inhibitor canagliflozin increased the risk of limb amputation by 2-fold compared with the placebo control [21]. The precise mechanism underlying limb amputation induced by SGLT2 inhibitor canagliflozin was unknown. However, pooled analysis of phase I–III trials of empagliflozin revealed that empagliflozin does not increase the risk of limb amputation [22]. Dehydration and a reduced vascular volume could be common risk factors for limb amputation caused by SGLT2 inhibition. We also observed a significant reduction of the ankle-brachial index caused by SGLT2 inhibitor ipragliflozin in patients with T2DM [23]. However, there is the possibility that the reduction of vascular thickening by empagliflozin, which was observed in our present study, could contribute to no induction of limb amputation caused by empagliflozin [22], but not canagliflozin [21], as a drug-specific effect among SGLT2 inhibitors.

In the present study, we treated diabetic mice with both linagliptin and empagliflozin, and observed vascular injury. Interestingly, the combined therapy caused an additive and significant reduction in neointima formation (Fig. 1A and B), suggesting that the combined treatment may reduce the risk of restenosis after coronary angioplasty and vascular thickness induced by vascular injury compared with single treatments. Furthermore, *in vitro* experiments confirmed additive attenuation of VSMC proliferation by linagliptin and empagliflozin (Fig. 3D). Similar to linagliptin in our previous report [15], empagliflozin decreased VSMC proliferation in a dose-dependent manner (Fig. 3B). However, empagliflozin did not decrease VSMC proliferation in low glucose medium (Fig. 3C), suggesting that inhibition of glucose uptake could be the mechanism by which empagliflozin attenuates VSMC proliferation. However, another group found that SGLT2 inhibition activates AMP-activated protein kinase and induces vascular relaxation [24]. Further mechanisms by which SGLT2 inhibition protects vascular functions should be examined in the future. In *db/db* mice, empagliflozin, but not linagliptin, reduced the blood glucose level (Fig. 2B and C), probably because severe hyperglycemia with glucose toxicity decreases GLP-1 and gastric inhibitory peptide receptor expression in pancreatic β cells [25], and linagliptin did not decrease the blood glucose level. Furthermore, in the present study, the reduction of neointima formation after vascular injury by linagliptin was lower and not significant compared with our previous report using non-diabetic mice [15], probably also because of glucose toxicity. It has been recently reported that hyperglycemia and glucose toxicity decreases GLP-1 receptor expression in vascular cells of *db/db* mice [26]. Accordingly, glucose toxicity reduces GLP-1 actions in not only pancreatic β cells, but also vascular cells. Therefore, the reduction in glucose toxicity by empagliflozin may enhance the vascular protective effect of linagliptin in the combined treatment.

In the present study, there is a limitation regarding the dosage of empagliflozin. Compared with a previous report administering empagliflozin (10 mg/kg/day) by oral gavage [27], we treated db/db mice with higher doseage of empagliflozin (30 mg/kg/day). In addition, because we employed combined treatment with empagliflozin and linagliptin, we could not examine dose-dependency of empagliflozin. An experiment with a lower dosage of empagliflozin should be performed in the future.

In conclusion, combined treatment with linagliptin and empagliflozin attenuated neointima formation after vascular injury in diabetic mice and VSMC proliferation *in vitro*.

## Funding

We do not receive any funding for the present study.

## Abbreviations

BrdU: bromodeoxyuridine
CV: Cardiovascular
DPP-4: dipeptidyl peptidase-4
GAPDH: glyceraldehyde-3-phosphate dehydrogenase
GTT: glucose tolerance test
ITT: insulin tolerance test
RASMC: rat aortic smooth muscle cells, RT reverse transcription
SGLT2: sodium glucose cotransporter 2
T2DM: type 2 diabetes mellitus
TUNEL: Terminal deoxynucleotidyl transferase-mediated dUTP nick end labeling
VSMC: vascular smooth muscle cells
